# Effectiveness of Pharmacist Interventions in Improving Medication Use in Hospitalised Older Patients Diagnosed With Cardiovascular Diseases: INFAR Before‐and‐After Study

**DOI:** 10.1111/jep.70190

**Published:** 2025-06-29

**Authors:** Romana Santos Gama, Luiz Carlos Passos, Welma Wildes Amorim, Renato Morais Souza, Marcio Galvão Oliveira

**Affiliations:** ^1^ Postgraduate Program in Pharmaceutical Services and Policies Federal University of Bahia Salvador Bahia Brazil; ^2^ Department of Internal Medicine, Postgraduate Program in Medicine and Health Federal University of Bahia Salvador Bahia Brazil; ^3^ Department of Health Sciences State University of Southwest Bahia, Vitória da Conquista Campus Vitória da Conquista Bahia Brazil; ^4^ Sarah Kubitschek Rehabilitation Center Brasilia Distrito Federal Brazil; ^5^ Multidisciplinary Institute in Health–Anísio Teixeira Campus Federal University of Bahia Vitória da Conquista Bahia Brazil

**Keywords:** clinical pharmacy, deprescribing, medication review, polypharmacy, potentially inappropriate medications, prescribing omission

## Abstract

**Objectives:**

This study aimed to assess the effectiveness of pharmacist interventions to reduce the omission of evidence‐based cardiovascular medication, as well as polypharmacy and promote the deprescribing of potentially inappropriate medications in hospitalised older patients diagnosed with cardiovascular diseases.

**Methods:**

This before‐and‐after study was conducted among patients aged ≥ 60 years (*n* = 319) at a cardiovascular hospital in Brazil. Pharmacists conducted medication reviews for these patients. The first prescription on hospital admission and that at discharge were collected and compared for prescribing omission, polypharmacy, and prescribing of potentially inappropriate medications.

**Results:**

The mean patient age was 68.9 (±6.2) years. The mean incidences of prescribing potentially inappropriate medications and omissions decreased from 0.90 at admission to 0.10 at discharge (*p* < 0.001) and from 0.65 to 0.30 (*p* < 0.001), respectively. The number of potentially inappropriate medications prescribed decreased significantly, from 291 at admission to 28 at discharge, reflecting a 90% reduction. Additionally, the mean number of medications prescribed decreased from 9.8 to 6.5 (*p* < 0.001).

**Conclusion:**

This study emphasises the role of medication reviews by clinical pharmacists in reducing polypharmacy, prescribing omissions, and inappropriate prescribing in older adults with cardiovascular diseases, demonstrating that targeted pharmacist interventions improve medication safety.

**Trial Registration:**

Registered on ClinicalTrials.gov (NCT04800900).

## 1 Introduction

Cardiovascular diseases (CVDs) are significant health problems in older adults [1]. Notably, they are a leading cause of death, accounting for 17.9 million deaths annually, among noncommunicable diseases, which cause 74% of global deaths [2]. People aged 65 years and older with CVDs experience a considerable reduction in ‘successful’ life expectancy, defined as a life with good health and functionality [3]. It has been projected that 70% of individuals over 70 will develop CVDs, with more than 66% also experiencing additional non‐cardiovascular health issues. Moreover, these individuals are likely to present with multiple chronic health issues that contribute to a substantial medication burden.

Although pharmacological treatments can markedly improve the functionality of physiological systems, they carry a substantial risk of harm [4]. More than half of all preventable harm in medical care worldwide is attributable to medication‐related issues [5]. The prescribing process is associated with the highest prevalence of preventable medication‐related harm [6]. Older patients and those in specialised or emergency care settings are particularly vulnerable because they are exposed to a high risk of preventable medication‐related harm, such as inappropriate prescribing, prescribing omission, and polypharmacy‐related harm. Consequently, integrating pharmacists' medication reviews is a promising strategy for improving the detection of such harm [7].

Studies have indicated that many geriatric patients receive medications without proper indications, at excessive doses, or for prolonged periods, whereas others may not receive the necessary treatment. These issues, characterised by overprescribing and prescribing omission, increase the risk of adverse health outcomes [8]. To mitigate these risks, medication reviews and deprescribing, i.e., the reduction or discontinuation of medications when their risks outweigh the benefits, should be integral to patient care [8]. Clinical pharmacists play a crucial role in this process because their extensive knowledge of medications and regular patient interactions enable them to optimise prescriptions, reduce polypharmacy, and improve overall clinical outcomes [9]. Evidence strongly supports pharmacists’ involvement in the care of hospitalised patients, highlighting their capacity to markedly improve patient safety and treatment effectiveness [8]. In this context, interventions conducted with the participation of clinical pharmacologists or hospital pharmacists (especially with adequately trained specialists) can significantly enhance medication review and the effectiveness of the deprescribing process [10].

This study was aimed at evaluating the effectiveness of pharmacist interventions to reduce the omission of evidence‐based cardiovascular medications as well as polypharmacy and promote the deprescribing of potentially inappropriate medications in hospitalised older patients diagnosed with cardiovascular diseases.

## 2 Methods

### 2.1 Procedures

#### 2.1.1 Study Design

INFAR (Effectiveness of Pharmaceutical Interventions in Hospitalized Patients; ClinicalTrials.gov identifier NCT04800900) was a before‐and‐after uncontrolled study. In this study, medication reviews were conducted by pharmacists at hospital admission and during the hospitalisation of the patients included in the study.

#### 2.1.2 Population

The INFAR study was conducted from March 2021 to January 2022 at the Ana Nery Hospital as a reference for CVD management in the public health system of Bahia, Brazil. The inclusion criteria were patients aged 60 years or older with a diagnosis of CVD who were hospitalised for more than 24 h. Patients who died in the hospital were excluded from the study because the study focused on evaluating admission and discharge prescriptions. Participants were recruited exclusively from the hospital's medical wards and invited to participate in the study after 24 h of hospitalization. Data collection encompassed the medications prescribed at both hospital admission and discharge.

#### 2.1.3 Outcomes

Omissions were identified using the START criteria translated into Portuguese and included 22 indicators of potential prescribing omissions in older adults, grouped by physiological systems [11]. Polypharmacy was defined as the prescription of 5 or more medications, and excessive polypharmacy as the prescription of 10 or more medications. The primary outcome of this study was potentially inappropriate prescription prevalence, assessed using the MPI Brasil application (app) [12]. This application was developed based on the Brazilian Consensus on Potentially Inappropriate Medications for Older People [13], aiming to validate the Beers and STOPP Criteria (2012 and 2006, respectively) contents to establish Brazilian‐specific criteria for older adults. In this study, we used the second version of the app, which incorporates the updates from the STOPP and Beers criteria (2014 and 2019, respectively). The MPI Brasil app is a comprehensive resource for evaluating potentially inappropriate medications and was designed to facilitate access to this information. It outlines the rationale for classifying a drug as potentially inappropriate and specifies exceptional situations in which a medication may not be considered potentially inappropriate. Furthermore, the app includes a convenient search function and suggests safer therapeutic alternatives, deprescribing strategies, and monitoring protocols for cases in which potentially inappropriate medication use is unavoidable [14].

Data on prescribing omissions, polypharmacy, and potentially inappropriate prescribing were collected twice, i.e., in the form of the first prescription 24 h after hospital admission and then the discharge prescription. One prescription per patient was analysed at each stage, ensuring that the same number of patients and prescriptions were included in both time points for comparison. Patient information and details were collected through interviews. Clinical data and prescriptions were obtained from medical records. The questionnaire and tools used for data collection were entered into the KoBoToolbox® online data collection platform.

#### 2.1.4 Variables of Interest

The age‐adjusted Charlson Comorbidity Index (ACCI) was chosen to associate the comorbidity burden because this index incorporates age into the Charlson Comorbidity Index to predict mortality and survival. The result is determined by the sum of the comorbidity weights plus an additional score for every 10‐year period starting at the age of 50 years [15]. The higher the ACCI score, the lower the estimated 10‐year survival, with scores of 1, 2, 3, 4, 5, 6, and ≥ 7 corresponding to survival rates of 96%, 90%, 77%, 53%, 21%, 2%, and 0%, respectively [15].

Cognitive impairment was assessed using the Mini‐Mental State Examination (MMSE), a screening tool for evaluating the progression of cognitive disorders, the scores of which range from 0 to 30. Cutoff points were adjusted according to participants’ educational levels, because the threshold for abnormality varies with educational attainment [16]. Daily activities and independence were evaluated using the adapted Katz Index of Independence in Activities of Daily Living, which analyses individuals’ abilities to perform daily tasks such as dressing, mobility, feeding, and attending to physiological needs [17].

#### 2.1.5 Sampling

To calculate the sample of prescriptions verified before and after the intervention, the following factors were considered: an expected pre‐intervention proportion of potentially inappropriate prescribing of 50%, an expected difference before and after the intervention of −30%, a significance level of 5%, and a test power of 80%. A 20% loss of prescriptions was also considered, resulting in a calculated sample size of 319 prescriptions. The samples were collected using a non‐probabilistic approach.

#### 2.1.6 Study Procedures

The hospital's pharmacy staff was trained to manage older patients more effectively and conduct medication reviews. Additionally, they were trained to use tools to improve the evaluation of appropriate prescribing for older patients, including a checklist with the START criteria translated into Portuguese, and the MPI Brasil app [14].

#### 2.1.7 Medication Review

The objective of the medication review was to assess and optimise the patient's entire medication regimen. Initially, all medications taken by the patients were identified, including prescription drugs, over‐the‐counter medications, herbal supplements, and vitamins. This information was collected through patient interviews, medical records, and occasional discussions with family members or caregivers. Subsequently, each medication was evaluated for appropriateness, effectiveness, safety, and potential adverse effects, considering the patient's health condition, age‐related changes, and other relevant factors. Special emphasis was placed on identifying medications that could exacerbate heart conditions or adversely interact with other treatments.

In addition, a checklist with the START criteria was used to identify any potential prescribing omissions, while the MPI Brasil app was used to detect potentially inappropriate medications [14, 17]. Based on this comprehensive evaluation, recommendations were made to attending medical teams. These recommendations included necessary interventions, such as the introduction of new therapies, deprescribing of unnecessary or potentially harmful medications, and dosage adjustments to optimise treatment efficacy and safety. Patients were monitored continuously throughout their hospitalisation, with adjustments made as needed to align with their evolving clinical conditions, ensuring that the interventions remained both safe and effective.

#### 2.1.8 Statistical Analysis

Data were analysed using SPSS Statistics for Windows, version 28.0 (IBM Corp., Armonk, NY). Sociodemographic and disease variables have been described as absolute and relative frequencies. Differences between the mean values for polypharmacy, potentially inappropriate medication, and prescribing omissions before and after the interventions were assessed using the Wilcoxon nonparametric test, because the Shapiro‐Wilk test indicated that the numerical variables were not normally distributed.

#### 2.1.9 Ethics Approval

This study was approved by the Ethics Committee on Research in Human Beings of Ana Nery Hospital, Salvador, Bahia (opinion number: 4.572.301). All the participants provided written informed consent.

## 3 Results

Of the 332 older patients interviewed, 13 were excluded because of death, resulting in a final sample of 319 individuals. The mean patient age was 68.9 (±6.2) years; 60.2% of the patients were male and 85.9% self‐identified as having black or brown skin colour. Based on the ACCI, a high comorbidity index (ACCI > 2) was identified in 71.5% of the patients. Of these patients, 40.1% had an estimated 10‐year survival rate of ≤ 53%. The mean number of comorbidities was 4.56 (±1.76), and the top five medical diagnoses were hypertension (80.6%), heart attack (47.6%), coronary artery disease (42%), heart failure (35.1%), and diabetes (34.5%) (Table 1).

**Table 1:** Sociodemographic and clinical characteristics of the study population (*n* = 319).

Characteristics	*N* (%)
Sex	
Male	192 (60.2)
Female	127 (39.8)
Literacy	
Illiterate	75 (23.5)
Literate	244 (76.5)
Skin colour	
White	45 (14.1)
Other (such as black and brown)	274 (85.9)
Smoking	
Nonsmoker	105 (32.9)
Smoker and former smoker	214 (67.1)
Alcohol consumption	
No	98 (30.7)
Yes	221 (69.3)
Cognitive impairment	
No	274 (85.9)
Yes	45 (14.1)
Poor self‐rated health	
No	90 (28.2)
Yes	229 (71.8)
Poor self‐rated memory	
No	226 (70.8)
Yes	93 (29.2)
ADL impairment	
No	285 (89.3)
Yes	34 (10.7)
Delirium	
No	314 (98.4)
Yes	5 (1.6)
Charlson Comorbidity Index age‐adjusted	
96% survival in 10 years	20 (6.3)
90% survival in 10 years	71 (22.3)
77% survival in 10 years	100 (31.3)
53% survival in 10 years	59 (18.5)
21% survival in 10 years	34 (10.7)
2% survival in 10 years	17 (5.3)
0% survival in 10 years	18 (5.6)
Medication use before admission	
No	34 (10.7)
Yes	285 (89.3)

Abbreviation: ADL, Activities of daily living.

A total of 3109 medications were prescribed during the initial prescription stage and 2060 medications were prescribed at discharge. During the initial prescription stage, the incidence of prescribing omissions was 47.3%; potentially inappropriate prescribing was identified in 79.3% of cases; and polypharmacy was noted in 98.5% of cases, 52.4% of which were classified as excessive polypharmacy. Among patients with polypharmacy (*n* = 314), 249 (79.3%) displayed at least one potentially inappropriate medication prescribed. Metoclopramide was the most frequently prescribed potentially inappropriate medication upon admission (220 prescriptions); however, the number of instances in which it was classified as such at discharge was 2, a significant decrease. Similarly, omeprazole was the most frequently prescribed potentially inappropriate medication at discharge (5 prescriptions); the number of instances in which it was classified as such at admission was 9 (Table 2).

**Table 2:** Top 10 potentially inappropriate medications prescribed.

Admission prescription (*n* = 291)		Discharge prescription (*n* = 28)	
Drug	*N* (%)	Drug	*N* (%)
Metoclopramide	220 (75.6)	Omeprazole	5 (17.9)
Clonazepam	28 (9.6)	Quetiapine	3 (10.7)
Omeprazole	9 (3.1)	Mineral oil	2 (7.1)
Quetiapine	6 (2.1)	Clonazepam	3 (10.7)
Mineral oil	5 (1.7)	Metoclopramide	2 (7.1)
Dimenhydrinate	4 (1.4)	Clonidine	3 (7.1)
Digoxin	2 (0.7)	Amitriptyline	4 (7.1)
Clonidine	1 (0.3)	Risperidone	5 (7.1)
Hydralazine	2 (0.3)	Furosemide	1 (3.6)
Carvedilol	3 (0.3)	Hydralazine	2 (3.6)

Furthermore, the number of potentially inappropriate medications decreased significantly from 291 at admission to 28 at discharge, reflecting an approximately 90% reduction through deprescribing. A total of 207 prescribing omissions were observed at admission and 97 at discharge. In both instances, the highest frequencies of omissions were associated with criteria involving the cardiovascular system (Table 3 and 4).

**Table 3:** Prescribing omissions at admission.

Drug	Clinical status	Number of patients with indication	Number of omissions	Frequency of omissions (%)
Metformin	Cases of type 2 diabetes or metabolic syndrome in the absence of renal dysfunction	110	105	95.5
Angiotensin‐converting enzyme inhibitors	After acute myocardial infarction	186	24	12.9
Beta‐blockers	Cases of stable chronic angina	194	16	8.2
Angiotensin‐converting enzyme inhibitors	Cases of chronic heart failure	105	10	9.5
Statins	Cases of peripheral or cerebral vascular disease when the patient's ‘functional status’ remains ‘independence in daily activities’ and life expectancy is > 5 years	242	9	3.7
Antiplatelet therapy	Cases of diabetes mellitus if 1 or more cardiovascular risk factors are present (hypertension, hypercholesterolaemia, or history of smoking)	112	9	8.0
Statins	Cases of diabetes mellitus with 1 or more cardiovascular‐associated factor	110	8	7.3
Aspirin or clopidogrel	Cases with a history of coronary artery disease, cerebral, or peripheral vascular disease with a sinus rhythm	208	7	3.4
Beta‐2 agonists or anticholinergic agents	Regular inhalation in cases of mild‐to‐moderate asthma or chronic obstructive pulmonary disease	12	5	41.7
Antidepressant drugs	Cases with moderate‐to‐severe depressive symptoms in the previous 3 months	8	4	50
Antihypertensive therapy	Cases in which the systolic blood pressure is consistently higher than 160 mmHg	265	2	0.8
Warfarin	Cases of chronic atrial fibrillation	53	2	3.8
Angiotensin‐converting enzyme inhibitor or angiotensin receptor blocker	Cases of diabetes with nephropathy, i.e., overt proteinuria, microalbuminuria on urinalysis (albumin level higher than 30 mg/24 h), or renal impairment.	37	2	5.4
Corticosteroids	Regular inhalation in cases of moderate‐to‐severe asthma or chronic obstructive pulmonary disease when the forced expiratory volume in 1 s is < 50%	7	2	28.6
Home continuous oxygen therapy	Cases in which chronic type 1 respiratory failure (pO_2_ < 60 mmHg; pCO_2_ < 48.75 mmHg) or type 2 respiratoryfailure (pO_2_ < 60 mmHg; pCO_2_ > 48.75 mmHg) has been documented	1	1	100
Calcium and vitamin D	Diagnosed cases of osteoporosis (radiological evidence or fracture due to previous fragility or acquired dorsal kyphosis)	1	1	100

Abbreviations: pCO_2_, partial pressure of carbon dioxide; pO_2_, partial pressure of oxygen.

**Table 4:** Prescribing omissions at discharge.

Drug	Clinical status	Number of patients with indication	Number of omissions	Frequency of omission (%)
Metformin	Cases of type 2 diabetes or metabolic syndrome in the absence of renal dysfunction	111	48	43.2
Angiotensin‐converting enzyme inhibitors	After acute myocardial infarction	193	9	4.7
Angiotensin‐converting enzyme inhibitors	Cases of chronic heart failure	113	8	7.1
Antiplatelet therapy	Cases of diabetes mellitus in which 1 or more cardiovascular risk factors are present (hypertension, hypercholesterolaemia, or a history of smoking)	112	6	5.4
Statins	Cases of diabetes mellitus with 1 or more cardiovascular associated factor	111	6	5.4
Statins	Cases of peripheral or cerebral vascular disease when the patient's ‘functional status’ remains ‘independence in daily activities’ and life expectancy is >5 years	247	6	2.4
Beta‐2 agonists or anticholinergic agents	Regular inhalation in cases of mild‐to‐moderate asthma or chronic obstructive pulmonary disease	11	4	36.4
Corticosteroids	Regular inhalation in cases of moderate‐to‐severe asthma or chronic obstructive pulmonary disease when the forced expiratory volume in 1 s is < 50%	7	3	42.9
Antidepressants	Cases with moderate‐to‐severe depressive symptoms during the previous 3 months	8	2	25
Aspirin or clopidogrel	Cases with a history of coronary artery disease, cerebral, or peripheral vascular disease with a sinus rhythm	220	2	0.9
Proton pump inhibitors	Cases of severe gastroesophageal acid reflux or peptic stricture requiring dilatation.	4	1	25
Warfarin	Cases of chronic atrial fibrillation	60	1	1.7
Antihypertensive therapy	Cases in which the systolic blood pressure is consistently higher than 160 mmHg	275	1	0.4

Additionally, the omission of metformin, a necessary prescription for patients with type 2 diabetes or metabolic syndrome, was the most common prescribing omission, with 105 instances at admission and 48 at discharge (Tables 3 and 4).

There were significant reductions in the rates of potentially inappropriate prescribing, prescribing omissions, and polypharmacy between admission and discharge (Table 5). Specifically, the incidence of potentially inappropriate prescribing was reduced from a mean of 0.90 (±0.7) at admission to 0.10 (±0.4) at discharge (*p* < 0.001). Similarly, the mean and maximum numbers of omissions decreased from 0.65 (±0.86) at admission to 0.30 (±0.65) at discharge (*p* < 0.001) and from 6 at admission to 4 at discharge, respectively. We observed a significant reduction in the proportion of prescription omissions (47.3% vs. 22.9%; *p* < 0.001), potentially inappropriate prescriptions (79.3% vs. 6.9%; *p* < 0.001), and polypharmacy (98.4% vs. 85.6%; *p* < 0.001) from admission to discharge. In addition, among the 273 patients with polypharmacy, 20 (7.3%) had at least one potentially inappropriate prescribed medication.

**Table 5:** Patients with polypharmacy, potentially inappropriate prescribing, and prescribing omission before and after pharmacist intervention.

	Mean		
	Admission	Discharge	*p*‐value
Omission	0.65 (±0.86)	0.3 (±0.65)	< 0.001
Potentially inappropriate prescribing	0.9 (±0.7)	0.1 (±0.4)	< 0.001
Number of prescribed drugs	9.8 (±2.5)	6.5 (±2.1)	< 0.001

Furthermore, the mean and maximum numbers of drugs prescribed decreased from 9.8 (±2.5) at admission to 6.5 (±2.1) at discharge (*p* < 0.001) and from 19 at admission to 14 at discharge, respectively.

## 4 Discussion

This before‐and‐after study evaluated the impact of pharmacist interventions on polypharmacy, potentially inappropriate medications, and prescribing omissions. Despite patients being cared for by cardiovascular specialists, polypharmacy, potentially inappropriate medications, and prescribing omissions were noted, highlighting the need for comprehensive medication reviews. By incorporating interventions, such as deprescribing and addressing omissions, we observed a reduction in the overall incidence of potentially inappropriate medication, prescribing omissions, and polypharmacy, underscoring the potential of clinical pharmacy to improve patient outcomes through targeted medication management.

One of the key strengths of this study is the use of an appropriate sample size drawn from a representative population, which enhanced the generalizability of the findings. Additionally, the use of patient medical records allowed for a more accurate assessment of inappropriate prescribing compared with reliance on patient surveys or pharmacy dispensing records. One notable strength is that this is the first study of its kind to evaluate the effectiveness of pharmacist interventions, specifically in older patients with CVDs, making this study unique.

However, this study has some limitations. It did not include an assessment of the acceptability of pharmacist interventions among physicians, which could have provided valuable insights into the practical implementation of these interventions. Additionally, potential confounders were not fully explored, such as interviews with prescribing physicians to understand the rationale behind potentially inappropriate medication or monitoring related adverse events. The impact of inappropriate prescribing on clinical outcomes was also not measured. Furthermore, the before‐and‐after study design has inherent methodological limitations, including the absence of blinding, potentially introducing bias. Moreover, we did not adjust for key confounding variables such as baseline disease severity, hospital stay duration, or treating clinical team‐related differences between the two periods.

Despite not being a primary objective, evaluating the effects of deprescribing at discharge could be beneficial. This is particularly relevant given that clinical condition relapse is among the concerns associated with deprescription and the prevalence of such relapse [18]. In addition, assessing these effects is important as it reportedly exerts a protective effect of medication review and deprescribing on rehospitalisation, especially within the first three months after hospital discharge [19].

Furthermore, this being a quasi‐experimental study, the design has inherent limitations, including the lack of random sampling and absence of a comparator group for participants receiving interventions. Additionally, the study design introduced the risk of unrecognised confounding factors and potential biases. Finally, owing to updates to the STOPP/START criteria, future research should explore whether similar results would be obtained using the latest version of the criteria. Unfortunately, the collected data could not be reanalysed using the third version of the STOPP/START criteria because the pharmacist team no longer had access to medical records. One study suggested that applying the STOPP/START criteria without clinical information may overestimate potentially inappropriate medication rates and underestimate prescribing omissions [20].

In our study, nearly all the individuals experienced polypharmacy during hospitalisation. Specifically, excessive polypharmacy was observed in 52.4% of the first prescriptions evaluated. A retrospective study involving older patients admitted to the cardiology department reported similar findings, with 95% of the patients experiencing polypharmacy and 69% experiencing excessive polypharmacy [21]. Additionally, a study conducted in 5 hospitals in Spain found polypharmacy and excessive polypharmacy rates of 93.8% and 58.8%, respectively [22]. The high incidences of polypharmacy and excessive polypharmacy observed in our study could be attributed to several factors.

First, the individuals presented a high comorbidity index, which, combined with prescribing omissions and the use of inappropriate medications, likely contributed to the increased incidence of polypharmacy [23]. Additionally, polypharmacy tends to increase during hospitalisation, because patients often require intensive treatment regimens to manage their acute conditions [24]. Moreover, older patients with CVDs are commonly prescribed multiple medications for primary and secondary prevention, symptom relief, slowing disease progression, and improving overall health outcomes [24]. Patients with a history of cardiovascular events are at an even greater risk of polypharmacy, given the need for comprehensive management to prevent recurrence [25].

Regarding the prescription of potentially inappropriate medication, 79.3% of the patients had at least 1 such prescription at baseline; this finding was consistent with those of similar studies conducted in Brazil (80.2%) and Spain (81.5%) [24, 26]. This high incidence was expected, given the high rates of polypharmacy and comorbidities in the study population. Specifically, in our study, the most frequently prescribed inappropriate medication on admission was metoclopramide, likely because of the prevalence of nausea and vomiting as symptoms in CVDs [27]. Notably, there was a considerable reduction in the number of potentially inappropriate medications from admission to discharge, indicating the effectiveness of deprescribing efforts. Omeprazole was the most frequently prescribed potentially inappropriate medication at discharge. However, the deprescribing of proton pump inhibitors such as omeprazole is often a gradual process because abrupt discontinuation can lead to rebound symptoms [28]. This reaction is an example of an adverse drug withdrawal event, which could typically be minimised, or even avoided, through a structured, patient‐centred deprescribing process involving planning, dose tapering, and close monitoring [18]. These findings highlight the importance of careful medication management to reduce the risk of adverse withdrawal effects when deprescribing potentially inappropriate medications.

Similar to other studies, in this study, there were frequent medication omissions during the admission and discharge stages, particularly concerning cardiovascular and endocrine treatments [29]. Almost half of the participants had at least 1 omitted medication upon admission. In addition, the omission of metformin was common at admission. At discharge, the same trends persisted, with cardiovascular and endocrine medications being the most omitted, with metformin remaining the most omitted medication. Metformin omission may be associated with the higher prescribing omissions observed in patients with diabetes [26]. The high frequency of omissions in these patients may be attributed to physicians’ intentions to minimise therapies for patients with CVDs owing to their comorbidities [30]. Moreover, efforts to reduce polypharmacy among older adults may result in prescribing omissions [29].

Medication reviews among older adults represent a critical component of healthcare management aimed at optimising pharmacotherapy and reducing medication‐related problems [31]. Our results indicate an improvement in prescribing practices at discharge after medication review and pharmacist interventions. This improvement was notable when this study was contrasted with another study, which reported an average of 11.6 (±4.5) medications prescribed for home use, without the implementation of pharmacist interventions [21]. The reduction in polypharmacy observed in our study can be attributed to the deprescribing of potentially inappropriate medications and the reduction in omissions.

Additionally, the proportion of patients taking at least 1 potentially inappropriate medication decreased significantly at discharge. This observation is in good agreement with a systematic review and meta‐analysis of 30 studies, most involving clinical pharmacologists or hospital pharmacists, in which 15 studies reported a reduction in potentially inappropriate medication at discharge following medication review and deprescribing interventions [10]. These finding reinforces the involvement of clinical pharmacists in significantly improving prescription quality by improving the deprescribing of inappropriate medications. Furthermore, these results are consistent with findings from systematic reviews and meta‐analyses, which indicate that pharmacist interventions can substantially reduce the incidence of potentially inappropriate medications in older adults [32].

Similarly, there was a significant reduction in drug omissions from admission. In contrast, a study conducted in New Zealand reported that the rate of prescribing omissions remained nearly unchanged from admission to discharge (40% vs. 39%) [33]. Similarly, a Brazilian study reported 39.6% omission at discharge [29]. None of these studies evaluated the impact of pharmacist interventions on prescribing omissions. This underscores the critical role of pharmacist oversight in ensuring that essential evidence‐based treatments for cardiovascular conditions are not omitted during care transitions, which is vital for improving clinical outcomes. The implementation of medication reviews and pharmacist interventions to address omissions likely contributed to optimised treatment regimens and improved patient safety.

## 5 Conclusion

This study highlights the role of clinical pharmacists in the management of medication therapy for older adults with CVDs. The significant reductions in polypharmacy, potentially inappropriate medications, and prescribing omissions underscore the effectiveness of targeted pharmacist interventions in improving medication safety and quality. Given the complexity of managing medicine in this population, integrating clinical pharmacists into healthcare teams is essential. Future research should focus on longitudinal outcomes to further assess the impact of these interventions on patient health beyond hospitalisation and explore their applicability across diverse healthcare settings. Ultimately, enhancing medication management through pharmacist involvement may lead to better health outcomes and improved quality of life in older adults with chronic health issues.

## Conflicts of Interest

The authors declare no conflicts of interest.

## Data Availability

The data that support the findings of this study are available on request from the corresponding author. The data are not publicly available due to privacy or ethical restrictions. Data cannot be shared for ethical/privacy reasons. The data underlying this article cannot be shared publicly owing to the sensitive nature of the topic and the risk of identification of participants. Data were stored on a shared, password‐protected channel of a Teams site accessible to all authors until the end of the project.
